# Functional genomic analyses highlight a shift in *Gpr17*‐regulated cellular processes in oligodendrocyte progenitor cells and underlying myelin dysregulation in the aged mouse cerebrum

**DOI:** 10.1111/acel.13335

**Published:** 2021-03-05

**Authors:** Andrea D. Rivera, Francesca Pieropan, Irene Chacon‐De‐La‐Rocha, Davide Lecca, Maria P. Abbracchio, Kasum Azim, Arthur M. Butt

**Affiliations:** ^1^ School of Pharmacy and Biomedical Science University of Portsmouth Portsmouth UK; ^2^ Department of Neuroscience Institute of Human Anatomy University of Padua Padua Italy; ^3^ Department of Pharmaceutical Sciences University of Milan Milan Italy; ^4^ Department of Neurology Neuroregeneration Medical Faculty Heinrich‐Heine‐University Düsseldorf Germany

**Keywords:** ageing, brain, drug discovery, GPR17, myelin, oligodendrocyte, oligodendrocyte precursor, remyelination

## Abstract

Brain ageing is characterised by a decline in neuronal function and associated cognitive deficits. There is increasing evidence that myelin disruption is an important factor that contributes to the age‐related loss of brain plasticity and repair responses. In the brain, myelin is produced by oligodendrocytes, which are generated throughout life by oligodendrocyte progenitor cells (OPCs). Currently, a leading hypothesis points to ageing as a major reason for the ultimate breakdown of remyelination in Multiple Sclerosis (MS). However, an incomplete understanding of the cellular and molecular processes underlying brain ageing hinders the development of regenerative strategies. Here, our combined systems biology and neurobiological approach demonstrate that oligodendroglial and myelin genes are amongst the most altered in the ageing mouse cerebrum. This was underscored by the identification of causal links between signalling pathways and their downstream transcriptional networks that define oligodendroglial disruption in ageing. The results highlighted that the G‐protein coupled receptor Gpr17 is central to the disruption of OPCs in ageing and this was confirmed by genetic fate‐mapping and cellular analyses. Finally, we used systems biology strategies to identify therapeutic agents that rejuvenate OPCs and restore myelination in age‐related neuropathological contexts.

## Graphical abstract

Ageing reduces the number of OPCs and their ‘stemness’. Genomic analysis resolved key mechanisms underpinning the age related decline in OPCs, most notably reduced expression of Gpr17 in a subpopulation of differentiation committed OPCs. Pharmacogenomics identified small molecules that have the potential to rejuvenate OPCs and promote myelination and repair.

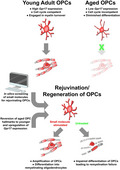

## 1 INTRODUCTION

Ageing in the brain is accompanied by a gradual decline in neuronal networking and synaptic plasticity which are needed for learning and cognitive function. Notably, neuronal numbers are largely unaltered in the ageing human brain (Fabricius et al., 2013; Pelvig et al., 2008). In comparison, there is evidence of gradual losses in oligodendrocytes and myelin in ageing and that these changes are key factors in cognitive decline and to decreased capacity for repair following pathology (Vanzulli et al., 2020). Sustaining myelin and oligodendrocytes throughout life is, therefore, critical and is the function of a reservoir of oligodendrocyte progenitor cells (OPCs) (Xiao et al., 2016). The underlying causes of myelin loss in ageing are unresolved, but there is increasing evidence that a major factor may be the decline in OPC regenerative capacity (Azim et al., 2017; Neumann et al., 2019). Hence, unravelling the fundamental changes in the ageing brain is a key strategy for developing new approaches to promote repair in neurodegenerative diseases, including Multiple Sclerosis (MS) and Alzheimer's disease (AD).

Transcriptomic studies have become increasingly important in understanding ageing processes in human and rodent oligodendrocytes (Azim et al., 2017, 2020; de la Fuente et al., 2020; Jäkel et al., 2019; Marques et al., 2016; Soreq et al., 2017). Here, using a combined transcriptomic and neurobiology approach we have identified essential oligodendroglial genes amongst the most dysregulated in the ageing mouse cerebrum, most notably *Gpr17*, which specifically decorates a subpopulation of differentiation committed OPCs (COPs) that are in transition to mature myelinating oligodendrocytes (MOLs) and react rapidly to brain pathology (Lecca et al., 2020). In addition, we determined specific cellular signalling pathways and transcriptional networks that characterise aged oligodendroglia. Finally, we used novel *in silico* pharmacogenomics strategies for the identification of therapeutic agents that stimulate the transcriptional networks for driving the regeneration of OPCs following demyelination and have therapeutic potential in MS and neurodegenerative diseases.

## 2 RESULTS

### 2.1 RNA‐seq transcriptome of the ageing mouse cerebrum

**FIGURE 1:**
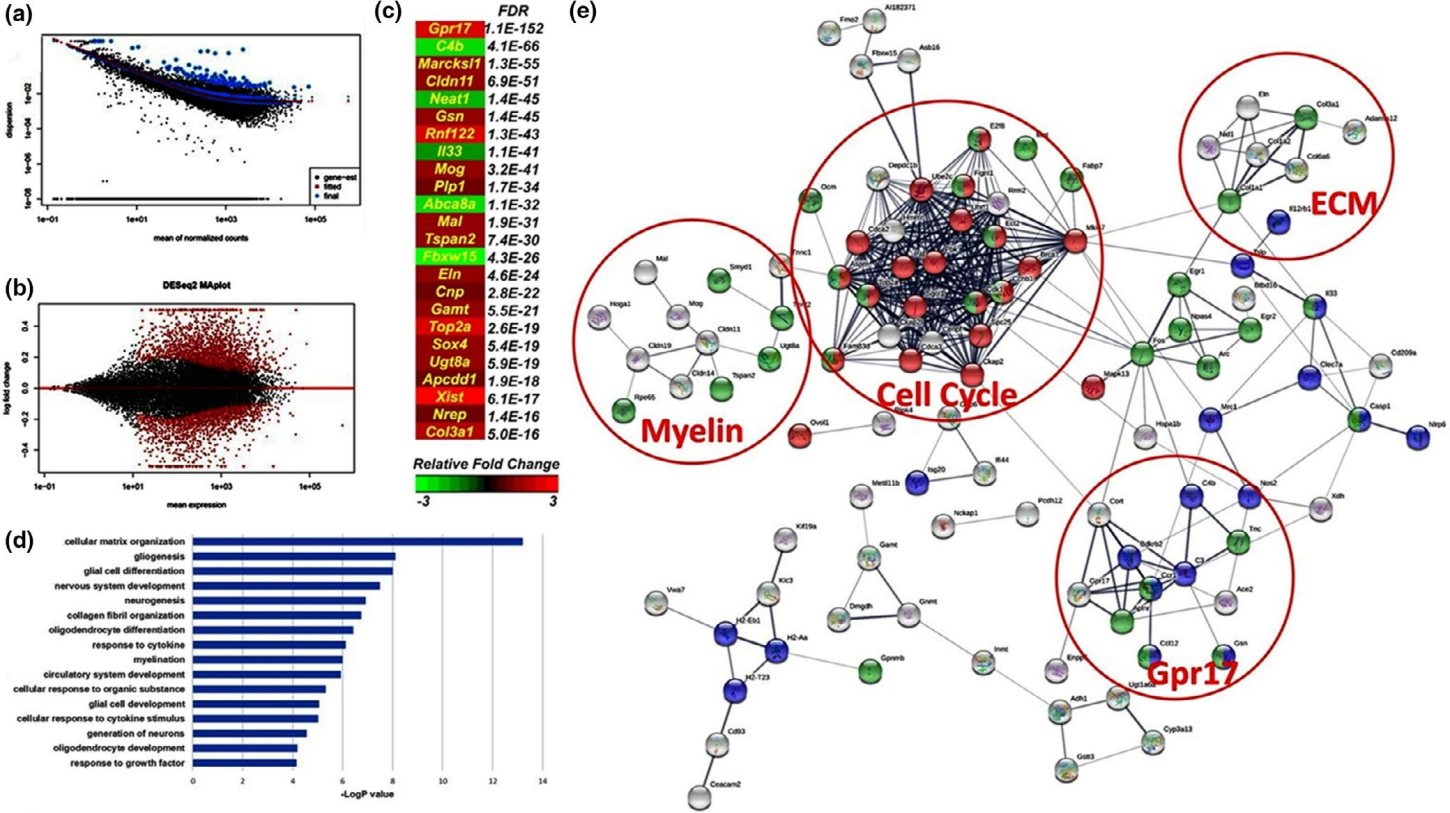
Transcriptomic characterisation of ageing‐induced genes in the brain. (a) QC of Datasets, analysis and dispersion plot of normalised mean gene counts. (b) MA plot illustrating the differential expression analysis and identified 1706 genes significantly altered between the two groups (FDR <0.01 or pADJ <0.01) using DEseq2 (V.1.4.2). (c) Heatmap of the most altered genes in the ageing cerebrum ranked by FDR values and colour intensity relative to log_2_ fold change. (d) Major ageing‐induced gene changes (threshold genes at FDR <0.05) represented by GO analysis revealing Extracellular Matrix (ECM) Organisation, Gliogenesis, Neurogenesis and Myelination among the most altered Biological Pathways. (e) Network analysis of predicted protein‐protein interaction performed with STRING (V.10.5) identified an alteration of the major processes and highlighting Cell Cycle (Red, FDR<0.0127), Cell Differentiation (Green, FDR<0.0307) and Inflammatory Response (Blue, FDR<0.0243, PPI Enrichment p‐Value <1.0e‐16)

The most prominent age‐related changes in the brain were explored by generating RNA‐seq profiles of dissected brain cerebrum from 1‐month‐old adult and 18‐month aged mice (Figure 1a‐c) and further investigating altered signalling and transcriptional networks using pathway analysis (ConsensusPathDB), functional protein–protein (STRING V10.5) interactions (Figure 1d, e) (Herwig et al., 2016; Szklarczyk et al., 2015), and protein‐chemical (STITCH v5.0) network analysis (Kuhn et al., 2008). A key finding was the predominance of oligodendroglial genes amongst the most significantly altered genes in the whole brain (Figure 1c). The most temporally regulated gene was *Gpr17*, which in the brain is expressed exclusively in a subset of rapidly reacting oligodendroglial cells, specifically in an intermediate stage between OPCs and terminally differentiated myelinating oligodendrocytes (MOLs) (Viganò et al., 2016). Single‐cell RNA‐seq of oligodendrocyte lineage cells (Marques et al., 2016) has identified the expression of Gpr17 in multiple clusters that can be collectively defined as ‘differentiation committed OPC’ (COPs) (Figure S1). In addition, the highest‐ranked genes altered in ageing were the major myelin‐related genes, *Mog*, *Plp1*, *Cnp* and *Ugt8a*, as well as the less well‐known myelin proteins *Cldn11* (Bronstein et al., 2000), *Tspan2* (Yaseen et al., 2017), and *Mal*, which regulates recruitment of PLP in myelin (Bijlard et al., 2016). These trends were verified by Gene Ontology (GO) analysis, which identified the main biological processes as those associated with Extracellular Matrix (ECM) Organisation and Gliogenesis/Differentiation, and specifically oligodendrocyte differentiation and myelination (Figure 1d). STRING Network Visualisation revealed that the most transcriptionally reshaped landscapes in the ageing cerebrum were associated with the control of cell cycle, and protein sub‐networks coupled to ECM remodelling and myelination, together with a transcriptional subnetwork associated with *Gpr17* (Figure 1e). The ECM plays a pivotal role in oligodendrocyte differentiation (Lourenço & Grãos, 2016) and increased stiffness of the ECM is related to age‐related deterioration of OPC function (Segel et al., 2019). Overall, these unbiased statistical analyses signify oligodendroglial genes as highly susceptible to age‐related changes in the mouse cortex.

### 2.2 Aged OPC transcriptional signature and myelination transcriptional networks

**FIGURE 2:**
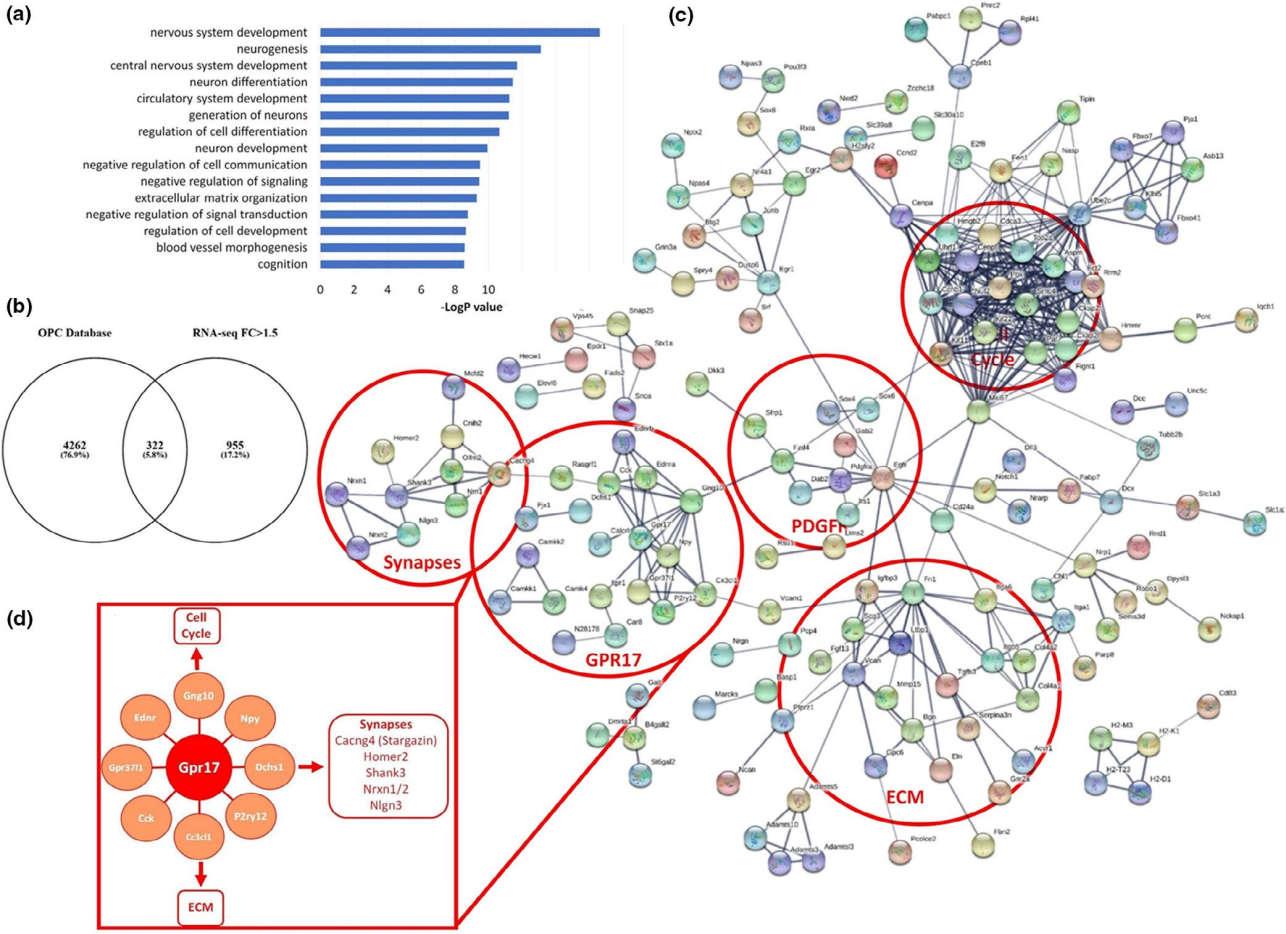
Age‐related transcriptional network alterations in OPCs. (a,b) GO analysis of OPC Biological Processes altered in ageing (a), from 322 core genes identified by meta‐analysis of RNA‐seq database of OPCs (b). (c) STRING analysis of the predicted interactions of OPC genes altered in ageing (PPI Enrichment *p*‐value <1.0e‐16); the circles represent groups of genes active along common pathways. (d) Highlighted *Gpr17* node and key interactions with Cell Cycle, Synapses and ECM nodes

To provide insight into the stage‐specific transcriptional signatures of aged OPCs (Figure 2) and MOLs (Figure S2), we performed a meta‐analysis of our RNA‐seq database against published datasets (Zhang et al., 2014). The results confirmed the most altered processes in aged MOLs were associated with myelination (Figure S2a,b), and at the core was *Egfr* (epidermal growth factor receptor) (Figure S2c), which has recognised importance in oligodendrocyte regeneration and myelin repair (Aguirre et al., 2007); interestingly, our analysis implicates dysregulation of a novel *Egfr*‐*Vinculin*‐*Gelsolin*‐*Cldn11* axis in the age‐related changes in myelination (Figure S2d), whereby the mechanosensitive function of EGFR is transduced by *Vcl* (Vinculin) and *Gsn* (Gelsolin) which, with *Cldn11* (claudin‐11), regulate the anchoring of the actin cytoskeleton to the ECM through integrins, that are essential for myelination (Bronstein et al., 2000; Zuchero et al., 2015). In aged OPCs, GO analysis demonstrated the highest‐ranked shifts in the cellular machinery were related to neural cell development, negative regulation of cell signalling and ECM organisation (Figure 2a). Functional Protein Interaction Network Analysis (STRING) uncovered the key aged OPC gene networks with the largest transcriptomic hub were related to the cell cycle operating downstream of signalling via the ECM and a *Pdgfra*‐*Gpr17* axis (Figure 2c). Further exploration of age‐induced OPC gene networks unravelled *Gpr17* as a multifactorial regulator, central to numerous pro‐oligodendroglial mechanisms, in addition to its known receptor function (uracil nucleotides and cysteinyl leukotrienes) in COPs, during the transition between OPCs and MOLs (Chen et al., 2009). Our analysis identified novel interactions between *Gpr17* and OPC differentiation, synaptic signalling and the ECM, together with prominent interactions between *Gpr17* and other G‐protein couple receptors, including *P2 yr12*, which mediates OPC‐ECM interactions that regulate differentiation (Dennis et al., 2012). *Gpr17* is an upstream hub for genes that encode for synaptic proteins in OPCs (Figure 2d), via the cell‐adhesion protein *Dchs1* (Dachsous Cadherin‐Related 1) and *Rasgrf1* (Ras Protein Specific Guanine Nucleotide Releasing Factor 1), which play essential roles in synaptic plasticity (Miller et al., 2013; Seong et al., 2015), and in the aged OPC gene network interconnect *Gpr17* with *Cacng4* (or Stargazin), together with the synaptic proteins *Shank3*, *Homer2*, *Nrxn1*/*2* and *Nlgn3*, which regulate synaptic targeting of AMPA receptors and bidirectional stabilisation of the pre‐ and post‐synaptic membranes (L. Chen et al., 2000; Dean & Dresbach, 2006; Shiraishi‐Yamaguchi & Furuichi, 2007). Notably, Stargazin targets AMPA receptors to the OPC cell membrane (Zonouzi et al., 2011), and AMPA receptors regulate OPC proliferation, differentiation and myelination (Larson et al., 2016). The aged OPC transcriptional signature locates *Gpr17* at the core of these OPC signalling networks that are most altered in the ageing brain.

### 2.3 Dysregulation of Gpr17 and oligodendrocyte differentiation in ageing

**FIGURE 3:**
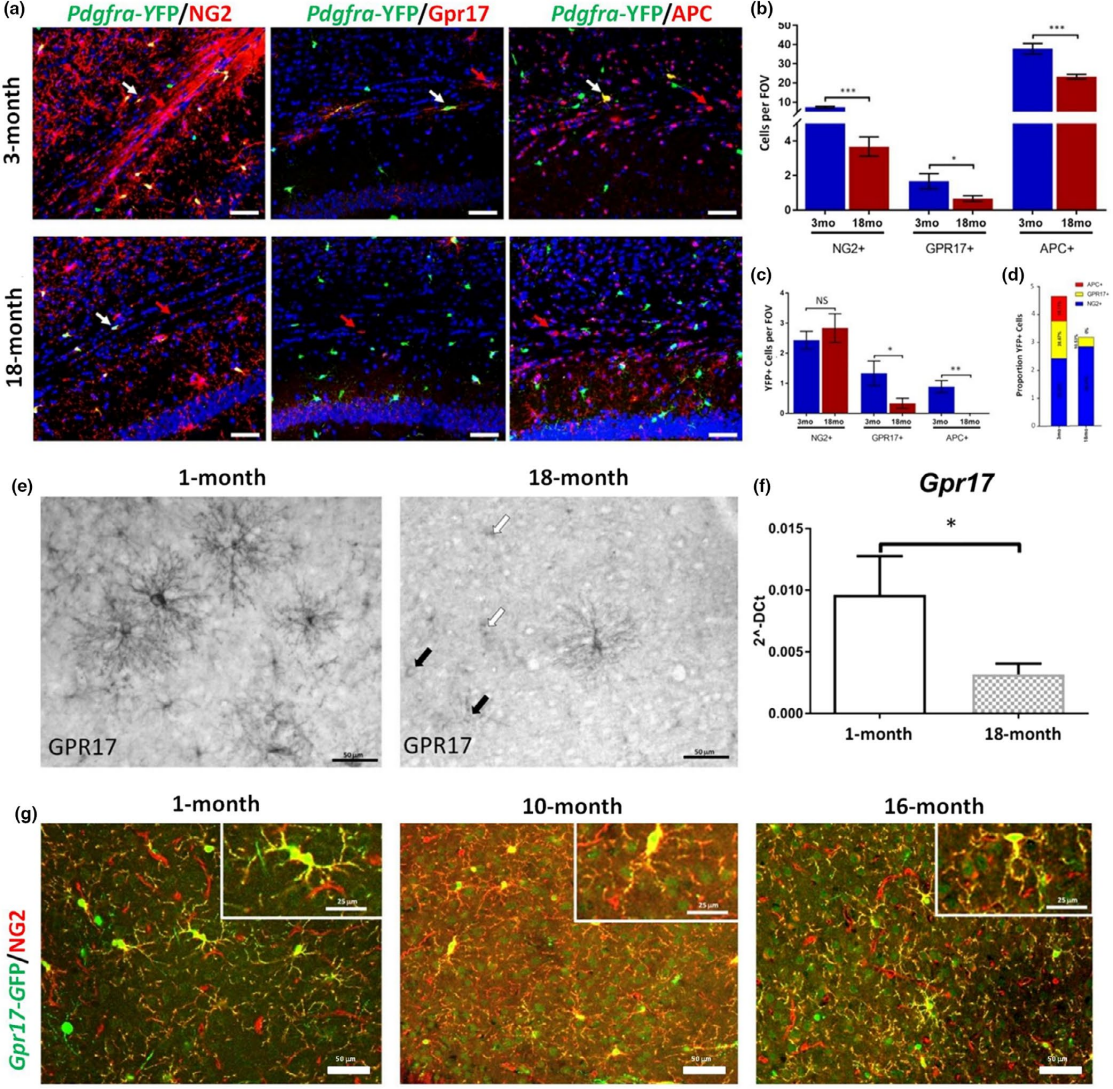
Dysregulation of Gpr17 and oligodendrocyte differentiation in the mouse cerebrum. (a) Immunostaining of *Pdgfra*‐CreERT2:Rosa26R‐YFP mice aged 3‐ (top panels) and 18‐months (lower panels) 10 days after genetic recombination, demonstrating a reduction in the number of NG2+ OPCs (left‐hand panels), Gpr17+ COPs (middle panels), and APC+terminally differentiated MOLs (right‐hand panels) in the cerebrum. Scale bars = 50 µm. (b) Quantification of total cell numbers per constant cerebral FOV showing a dramatic loss of NG2+ OPCs and Gpr17+ COPs, together with a significant decrease in APC+MOLs (n = 3 mice per group; *p < 0.05, ***p < 0.001, unpaired *t* tests). (c, d) Fate‐mapping of *Pdgfra*‐YFP+OPCs in defined differentiation stages, expressed as total number of cells per constant cerebral FOV (c) and as a proportion of the total number of YFP+cells (d), illustrating a marked decline in OPC differentiation into Gpr17+ COPs and a complete loss of cells differentiating into APC+MOLs over this period (n = 3 mice per group; *p < 0.05, **p < 0.01, unpaired *t* tests). (e) Chromogenic characterisation of Gpr17 expression in 1‐ and 18‐month‐old cerebrum in wild‐type C57BL/6 mice, indicating a loss of Gpr17+ expression in ageing; Gpr17 densely decorates somata and processes at 1‐month, whereas at 18‐month Gpr17+ COPs are either dimly immunostained (black arrows), or in many cases, only the cell somata are immunostained (white arrows); scale bars = 50 µm. (f) qPCR quantification of *Gpr17* expression in young and aged cerebrum; data are expressed as 2‐dCt (n = 3 mice per group; *p < 0.05, unpaired *t*‐test). (g) Fate‐mapping of Gpr17+/GFP+COPs immunolabelled with NG2 for all OPCs in the cerebrum of *Gpr17*‐iCreERT2xCAG‐eGFP mice 10 days after recombination; immunostaining shows a gradual age‐related decline in the density of OPCs and COPs (scale bars = 50 µm in main panels and 25 µm in insets)

To investigate how ageing OPC regulatory networks are translated into cellular changes, we examined substages of the OL lineage in the *Corpus Callosum* (CC) in Pdgfra‐CreERT2:Rosa26R‐YFP and Gpr17‐iCreERT2xCAG‐eGFP mice (Figure 3). First, Pdgfra‐CreERT2:Rosa26R‐YFP mice aged 3‐ and 18‐months were injected with tamoxifen twice a day for 5 days to induce YFP expression in OPCs. After 10 days following genetic recombination, immunostaining was performed for NG2, Gpr17 and APC to identify the key stages between OPCs, COPs and terminally differentiated MOLs (Figure 3a). Cell counts indicated that at 18‐months there were significant overall decreases in NG2+ OPCs and Gpr17+ COPs, together with a significant decrease in APC+ MOLs (Figure 3b). Fate‐mapping of Pdgfra‐YFP+ cells over the two‐week experimental period shows a marked decline in differentiation of Pdgfra‐YFP+ OPCs into Gpr17+ COPs, and a complete absence of subsequent differentiation into APC+ MOLs (Figure 3c). This is illustrated further by expression of YFP+ cells in each OL stage as a proportion of total YFP+ cells, which indicates a differentiation block of aged OPCs into COPs expressing Gpr17+ that regulates the transition from OPCs to MOLs (Viganò et al., 2016). To examine this further, we used chromogenic immunostaining (Figure 3e), together with qPCR to confirm *Gpr17* mRNA is significantly and markedly decreased in ageing (Figure 3f), and inducible expression of eGFP in Gpr17+ COPs (Figure 3g); this is supported by a concurrent report that Gpr17 protein and mRNA are reduced in ageing (de la Fuente et al., 2020). Chromogenic immunolabelling is exceptionally sensitive and demonstrates that in the ageing brain, few cells exhibit the dense immunostaining of cellular processes that is characteristic of younger brain (Figure 3d), and instead, COPs are either dimly immunostained (Figure 3e, black arrows) or in many cases, the cell somata alone are immunopositive (Figure 3e, white arrows). Chromogenic labelling shows Gpr17+ COPs persist in the ageing brain, and this was confirmed using tamoxifen‐inducible Gpr17‐eGFP mice (Figure 3g), but the number of COPs expressing Gpr17 and the level of expression in individual cells are markedly reduced. Overall, the results demonstrate that dysregulation of Gpr17 is central to age‐related disruption of OPCs and their differentiation into MOLs.

### 2.4 Identification of small molecules to rejuvenate aged OPCs

**FIGURE 4:**
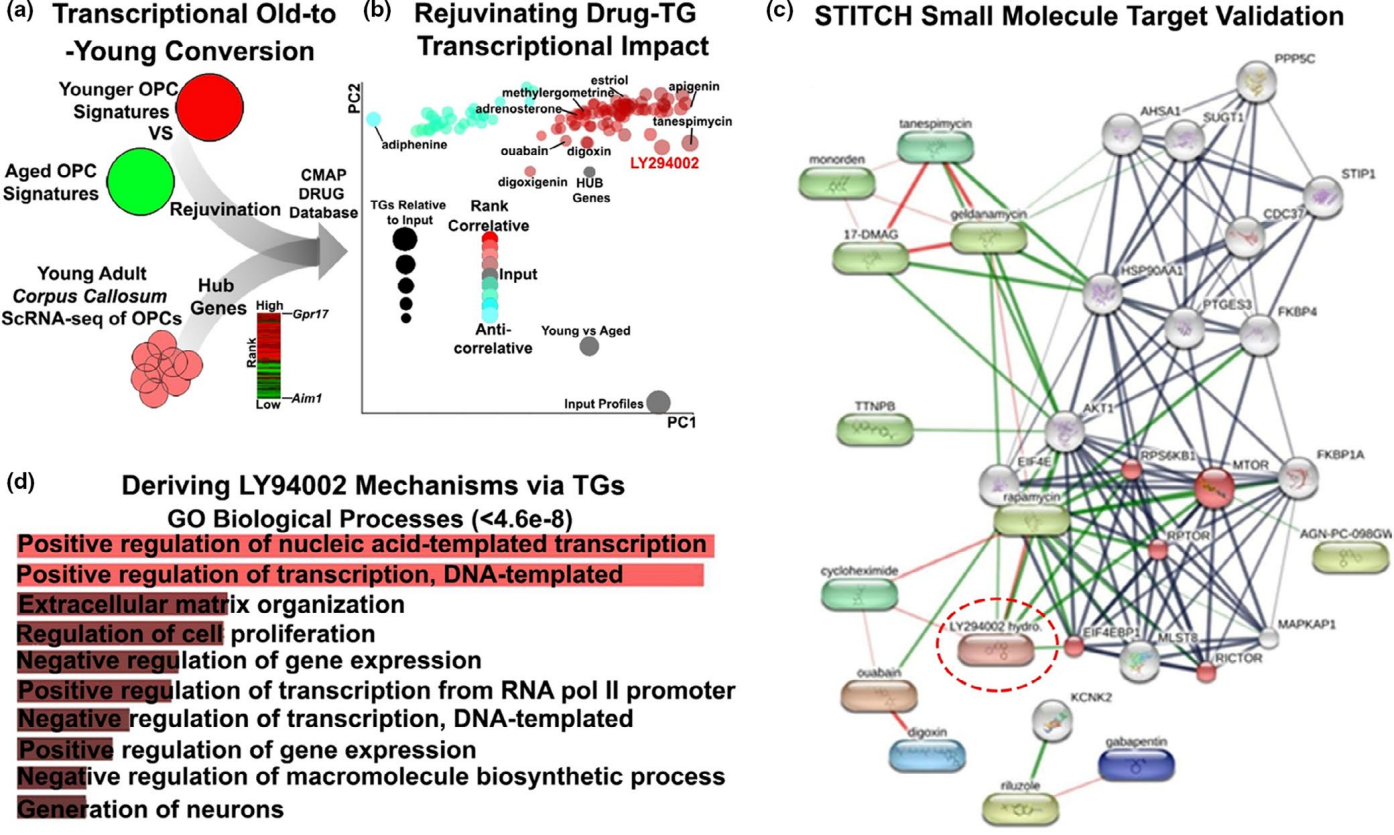
Pharmacogenomic identification of therapeutic agents for rejuvenating OPCs in ageing contexts. (a) Overview of the meta‐analyses performed for assembling transcriptional signatures using datasets generated in the present study and singlecell datasets of young OPCs for the detection of master regulators; combined gene lists were interrogated via the CMAP database. (b) Visualisation of obtained small molecules in a principle component plot by their target genes (TGs) reflected in the size of points and coloured using Pearson's correlation scores. (c) STITCH protein target analysis of pharmacogenomically‐derived small molecules predicted to rejuvenate OPCs, highlighting LY294002 operating via the mTOR pathway (Red, FDR<9.3e‐06) (PPI enrichment *p*‐value 3.11e‐16). (d) Biological Processes of LY294002 target genes (TGs) using the Enrichr webtool

Both the transcriptomic and fate‐mapping/immunohistochemical findings demonstrate that disruption of OPCs and MOLs are major factors in the ageing brain. We used the SPIED/CMAP database to identify small molecules that recapitulate transcriptional changes in younger OPCs (Figure 4), as previously described (Azim et al., 2017; Rivera & Butt, 2019). We interrogated the gene sets for young adult and aged OPCs generated above, together with previously curated single‐cell RNA‐sequencing gene sets of young adult OPCs. In this strategy, young adult OPC‐core genes were transformed to co‐expression hub genes against drug connectivity mapping databases (Williams, 2013), that highlight master regulators and identified *Gpr17* as the most highly correlated hub (Figure 4a), which fully validates the genomic and neurobiological data presented above. We then used two distinct approaches to identify small molecules that have the potential to rejuvenate aged OPCs, by interrogating the core OPC genes across the entire SPIED/CMAP database, presented in Figure 4b as a dimensionality reduction plot, in addition to a STRING chemical‐protein target analysis of all OPC genes differentially expressed in the ageing brain (Figure 4c). Significantly, these two separate approaches identified the same small molecules with the potential to specifically rejuvenate ‘stemness’ in aged OPCs (Figure 4b,c), and none of these small molecules were predicted to act on MOLs (Figure S3). In OPCs, a number of cardiac glycosides (digoxin, digoxigenin and ouabain) were highlighted as having OPC rejuvenation potential by regulation of mTOR signalling (Figure 4b,c). The small molecule with the highest number of associated target genes was LY294002, which was at the centre of the OPC rejuvenating drug network (Figure 4b,c), and is a known modulator of PI3 K‐Akt‐mTOR signalling, a key regulator of OPC differentiation and myelination (Ishii et al., 2019). Analysis of the biological processes of LY294002 target genes (TGs) in OPCs, using the Enrichr webtool (see Experimental Procedures), identified positive regulation of transcription, as well as ECM interactions and regulation of cell proliferation, as key mechanisms of action LY294002 in OPCs (Figure 4d). These analyses highlighted LY294002 as a potential therapeutic strategy for rejuvenating OPCs in the ageing brain.

### 2.5 The small molecule LY294002 identified by *in silico* pharmacogenomics promotes oligodendrocyte regeneration and remyelination in older mice

**FIGURE 5:**
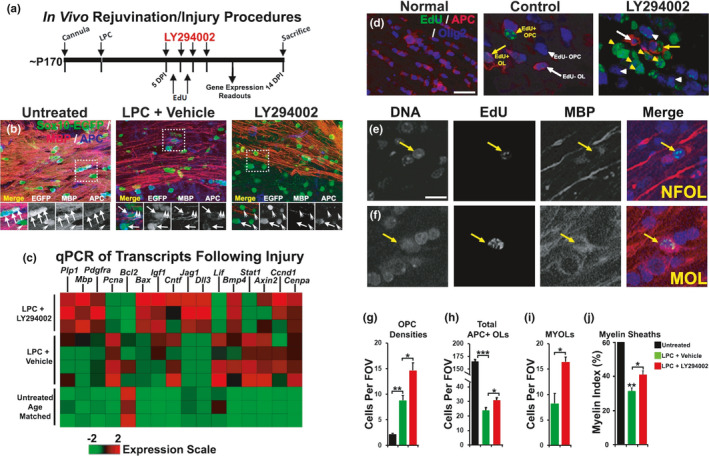
LY294002 rejuvenates OPCs following demyelination in older mice. (a) *In vivo* demyelination procedures, EdU injections, sampling for microdissected CC for qPCR and histology readouts. (b) Flattened confocal z‐sections of Sox10‐EGFP mice of 10 µm thickness, immunostained for myelin with MBP and differentiated OL somata with APC. (c) Heatmap of genes measured by qPCR of microdissected CC at 10DPI in untreated, lysolecithin (LPC)+vehicle and LPC+LY294002. (d) Flattened confocal z‐sections (10 µm thickness) of wild‐type mice CC at 14 DPI immunostained for Olig2 for OL lineage cells, APC and EdU for differentiated oligodendrocytes. (e,f) Higher magnification to illustrate non‐myelin forming oligodendrocytes (NFOLs) and myelin‐forming oligodendrocytes (MYOLs) that had incorporated EdU earlier in the lineage. (g–j) Quantification of Sox10‐EGFP+/APC‐ cells and PDGFRα+ OPCs in the CC (g), total numbers of APC+OLs (h), mature myelin‐forming MYOLs (i), and the myelin index (i); ns = no significance; *=*p* < 0.05; **=*p* < 0.01; ***=*p* < 0.001; unpaired students *t* tests

To assess the effects of LY294002 on the capacity of adult OPCs to regenerate MOLs, we analysed the remyelination power of OPCs *in vivo* following intraventricular infusion of the demyelinating agent lysolecithin (LPC) in Sox10‐EGFP mice, which identifies oligodendroglial cells at all stages of differentiation (Azim & Butt, 2011). LPC (2%), or sterile vehicle in controls, were administered by intraventricular injection in mice aged on average 6 months, which was selected because this age is a point of inflection, after which there is a decline in the rate and overall extent of remyelination, owing to diminished OPC regenerative capacity (Crawford et al., 2016). At 5 days post‐injection (DPI), LY294002 was administered by osmotic pump to provide a final concentration of 2 µM in the CSF, calculating for the dilution effect in the ventricular volume (Azim & Butt, 2011). The cell proliferation marker EdU was administered at 5 and 6 DPI, for fate‐mapping of newly formed OLs (NFOLs), and brains were analysed at 10 and 14 DPI (Figure 5a). Immunolabelling for MBP confirmed evident demyelination in the CC at 14 DPI following LPC injection, together with an apparent decrease in the overall number of Sox10+ oligodendroglia (OPCs and MOLs) and APC+ MOLs, compared to controls, and these were evidently improved by LY294002 treatment (Figure 5b). These effects of LY294002 were confirmed by qPCR of microdissected CC from mice treated with LPC or LPC+LY294002, compared to age‐matched untreated mice (Figure 5c). The results demonstrate that the major OPC and MOL transcripts *Pdgfra*, *Plp1* and *MBP* were all increased significantly in LY294002, compared to LPC and untreated controls (Figure 5c). In addition, LY294002 had pro‐oligodendroglial and anti‐inflammatory effects compared to LPC treatment, for example, *Igf1* and *Bmp4* are increased, whereas *Lif* and *Stat1* are decreased (Figure 5c). Finally, we analysed the changes in distinct oligodendrocyte differentiation stages by immunolabelling for the pan‐oligodendroglial marker Olig2, together with EdU and APC (Figure 5d), to identify and quantify total numbers of Olig2+/APC‐ OPCs and Olig2+/APC+ MOLs (Figure 5d,g,h). In addition, MBP immunostaining in MOLs is restricted to the myelin sheaths, whereas MBP is also expressed in the somata of NFOLs (Figure 5e,f, arrows), and the former were prevalent in demyelinated lesions following treatment with LY294002 (Figure 5i). To assess more precisely the level of remyelination, we used the myelin index (MI) which is a measure of the numerical density of myelin sheaths that cross the z‐plane in the CC (Figure 5j), as previously detailed (Azim et al., 2012). The data demonstrate that at 14DPI, compared to controls, there was a significant increase in OPCs and decreases in MOLs and the MI within demyelinated lesions following LPC (Figure 5g–j), consistent with the reported loss of oligodendrocytes and recruitment of OPCs in this model. Notably, treatment with LY294002 significantly increased the numerical density of OPCs, compared to controls and LPC treatment, as well as significantly increasing MOLs, the MI, and the regeneration of NFOLs compared to LPC treated mice (Figure 5h–j). The *in vivo* effects of LY294002 validate the *in silico* pharmacogenomic analysis that identifies multiple small molecules with the potential to rejuvenate OPC stemness and promote remyelination and repair.

## 3 DISCUSSION

Age‐related changes in myelination are proposed to be a major factor in cognitive decline and are implicated in myelin loss in AD and the ultimate failure of remyelination and repair in MS, although the underlying mechanisms remain unclear (Neumann et al., 2019; Vanzulli et al., 2020). Our differential transcriptomic analysis demonstrates that oligodendroglial genes are amongst the most significantly dysregulated in the mouse cerebrum in natural ageing. Notably, our results highlight *Gpr17* as a major factor affected during oligodendrocyte degeneration in the ageing brain. Moreover, we unravelled key transcriptional networks and signalling pathways that are central to age‐related dysregulation of myelin turnover. Finally, we identified specific pro‐oligodendroglial small molecules that rejuvenate OPC stemness and promote remyelination and repair. This study unravels new mechanisms in natural ageing and in neurodegenerative diseases.

### 3.1 Oligodendroglial transcriptomic networks are significantly altered in ageing

Transcriptomic analysis identified dysregulation of multiple biological processes that are critical for normal brain function, most significantly multiple processes that are associated with dysregulation of the ECM and myelination, together with oligodendrogliogenesis. Notably, these processes are interrelated and the ECM plays a major role in the biomolecular and biomechanical regulation of OPC stemness (Segel et al., 2019). Our data implicated an Egfr‐vinculin‐gelsolin‐Claudin11 axis at the centre of oligodendroglial networks that are altered in ageing, which provides a potential mechanism by which the mechanosensitive function of Egfr (Müller‐Deubert et al., 2017; Tschumperlin et al., 2004) is transduced via vinculin and gelsolin to regulate cell/integrin/ECM interactions and control oligodendroglial cytoskeleton dynamics and cellular spreading (Hagen, 2017; Rübsam et al., 2017; Sehgal et al., 2018). Several lines of evidence show that Egfr promote oligodendrocyte regeneration and myelin repair (Aguirre et al., 2007), and our findings indicate Egfr signalling is pivotal to multiple transcriptional networks and signalling pathways that regulate age‐related changes in oligodendrocytes.

### 3.2 Disruption of Gpr17+ OPCs in ageing

Changes in OPCs were a major hallmark of the ageing brain and specifically dysregulation of Gpr17. In the adult healthy brain, OPCs normally proliferate at very low levels, but mediate rapid repair responses to injury with increased proliferation and differentiation to mature myelinating cells (Psachoulia et al., 2009). Interestingly, Gpr17 specifically decorates a subset of OPCs responsible for such rapid reaction to damage (Viganò et al., 2016). These findings are in line with studies reporting heterogeneity of OPCs and identifying several progenitor/immature oligodendrocyte stages where Gpr17 is most highly expressed in ‘differentiation committed OPC’, or COPs (Marques et al., 2016) (Figure S1), suggesting that ageing specifically affects OPC subpopulation(s) conferring plasticity to the brains regenerative responses. It is significant that changes related to neural cell differentiation are the top processes affected in aged OPCs, and the largest transcriptomic hub is related to the cell cycle, consistent with evidence OPC self‐renewal declines with ageing (Young et al., 2013), which underpin the striking reduction in OPC numbers observed in the 18‐month brain and age‐associated loss of plastic responses of OPCs and COPs to insults and damage. Interestingly, our transcriptomic analysis of aged OPCs identified a hub of genes that encode for the synaptic proteins Stargazin, Shank3, Homer2, Neurexin1/2 and Neuroligin3, which are all central to glutamatergic synapses that regulate OPC proliferation and differentiation (Chacon‐De‐La‐Rocha et al., 2020; Chen et al., 2018; Larson et al., 2016). Myelination of new neuronal connections is dependent on neuronal activity and failure of OPCs to generate new oligodendrocytes retards myelination and learning ability (Gibson et al., 2014; McKenzie et al., 2014; Wake et al., 2011; Xiao et al., 2016). On the other hand, OPCs are known to support neuronal integrity and function by providing neurons with lactate, whose production is, in turn, strictly dependent on OPC energy metabolism. The previous demonstration that Gpr17 regulates lactate secretion by OPCs on neurons (Ou et al., 2019) suggests that the massive reduction of Gpr17 expressing OPCs during ageing could contribute to age‐associated neuronal deterioration and learning deficits also via this additional mechanism.

### 3.3 Dysregulation of Gpr17 expression in aged OPCs

The transcriptomic and cell biological data all point to Gpr17 as being central to age‐related changes in the brain. As mentioned above, Gpr17 is specifically expressed by a subset of OPCs that are normally quiescent but rapidly react to insults such as brain ischaemia (Viganò et al., 2016), suggesting that they may serve as a ‘reservoir’ of cells specifically devoted to repair purposes. Although these cells fail to repair damage in excessive inflammatory milieu (Bonfanti et al., 2020), under ‘permissive conditions’ (i.e., in the presence of low inflammation levels), they generate myelinating oligodendrocytes and ameliorate damage (Coppolino et al., 2018). In line with data showing that increased inflammation in the aged brain is associated with reduced repair abilities, our data indicate there is a marked reduction of NG2+ OPCs and MOL at 18 months, consistent with a recent study indicating aged OPCs lose their stem cell characteristics (Neumann et al., 2019). More importantly, our fate‐mapping study shows for the first time that there is a marked decline in replenishment of Gpr17+ COPs from OPCs, with a subsequent loss of MOLs. Gpr17 can be activated by uracil nucleotides and cysteinyl leukotrienes, whose brain levels increase upon damage, although under excessive inflammatory conditions, such as those also found in the aged brain, Gpr17 can be pathologically activated by oxysterols and stromal cell‐derived factor‐1 (SDF‐1) (Fumagalli et al., 2011; Parravicini et al., 2016). At early differentiation stages, Gpr17 delays OPCs differentiation, via activation of Gα_i/o_ and inhibition of cAMP‐PKA (Simon et al., 2016), whereas at later maturation stages, Gpr17 removal from the membrane via ligand‐induced desensitisation by G‐protein receptor kinase phosphorylation is necessary for terminal differentiation of COPs (Daniele et al., 2014; Fumagalli et al., 2011). By binding to Gpr17, inflammatory molecules could disrupt stage‐dependent Gpr17 regulatory mechanisms, thus resulting in impaired COP terminal maturation and myelination. Since Gpr17 is expressed on the cell membrane, and thus amenable for pharmacological manipulation, we envisage that novel selective molecules directly acting at the Gpr17 receptor level could revert ageing associated effects on myelination (Parravicini et al., 2020). Notably, in line with the above findings and with Gpr17 function as a sensor for brain damage (Lecca et al., 2008), antagonism of Gpr17 has a rejuvenation effect in the ageing brain (Marschallinger et al., 2015). Our chromogenic immunohistochemical data and Gpr17 fate‐mapping in Cre‐Lox mice demonstrate major disruption of Gpr17 at both the mRNA and protein level in aged OPCs. In addition, we identified novel interactions in *Gpr17* that are altered in ageing OPCs, with a prominent interaction with *Gng10* (G‐Protein Subunit Gamma 10), which links *Gpr17* to both OPC proliferation and negative regulators of OPC differentiation, namely *Pdgfra*, *Sox 4*, *Sox6* and *Egfr* (Baroti et al., 2016; Braccioli et al., 2018; Ivkovic et al., 2008). Our data identify a pivotal role for Gpr17 dysregulation in the ageing brain and the decline in OPC capacity to regenerate oligodendrocytes.

### 3.4 Pharmacogenomic screening identifies LY294002 as a therapeutic target for stimulating OPCs in the context of remyelination in older mice

We employed two separate pharmacogenomic approaches for determining: (a) the most optimal therapeutic agents for enhancing the densities of OPCs in the CC and their terminal differentiation into oligodendrocytes; (b) small molecules capable of reshaping aged transcriptional networks into their younger counterparts where their efficiency for myelin generation is more pronounced. Small molecules obtained in our analysis included those that target mTOR regulated cellular processes, including lipid metabolism, nucleotide synthesis and translation (Figlia et al., 2018), and, more specifically for this study, Gpr17 signalling and OPC maturation (Fumagalli et al., 2015; Ren et al., 2012). The PI3K/Akt/mTOR signalling inhibitor LY294002 was identified as the most potent small molecule for shifting the transcriptional hallmarks of aged OPCs into those characteristic of younger OPCs. LY294002 target genes in rejuvenating OPCs included ECM reorganisation, which we show above is dysregulated in aged OPCs. Importantly, we demonstrate that LY294002 promotes regeneration of OPCs and oligodendrocytes *in vivo* following demyelination induced by the toxin lysolecithin in 6‐month‐old mice, at which age the pace of remyelination is markedly impaired compared to younger adults (Crawford et al., 2016; Kazanis et al., 2017). These results *in vivo* validate the *in silico* pharmacogenomic data and demonstrate that small molecules identified using this approach have considerable potential in reversing the decline in OPC function in ageing and promoting remyelination and repair, likely via multiple effects that may also include Gpr17 regulation. In this study, we did not specifically assess the rejuvenating effects of ligands directly acting on Gpr17 (Parravicini et al., 2020), which will represent the focus of future studies.

### 3.5 Conclusions

Our unbiased transcriptomic analysis identified oligodendroglial genes amongst the most altered in the aged mouse cerebrum, highlighting Gpr17 as a major factor in the disruption of the regenerative capacity of OPCs and decline in myelination. Unravelling the key transcriptional networks and signalling pathways that are central to age‐related dysregulation of OPCs, enabled us to pharmacogenomically stimulate OPC rejuvenation in the context of remyelination. Finally, it should be noted that changes in neuroglia appear to be a general feature of ageing, with evidence that astrocytes, microglia and OPCs undergo cellular atrophy, with a concurrent disruption of function in the course of normal ageing (Streit et al., 2004; Vanzulli et al., 2020; Verkhratsky et al., 2020). These studies provide a framework for future investigations in the field for targeting cellular mechanisms underlying the decline in glial plasticity and highlight the power of systems biology tools for counteracting the age‐related decline in regenerative capacities and pathology.

## 4 EXPERIMENTAL PROCEDURES

### 4.1 Animals and tissue

All animal studies were performed in accordance with international law (European law Dir. 2010/63/UE) and national laws (UK Animals Scientific Procedures Act, 1984; Italian law DL n. 26, 4th March 2014). All procedures were reviewed and approved by the local ethical review bodies of the Universities of Portsmouth and Milan, with appropriate UK Home office Project Licence and the Italian Ministry of Health (authorisation 473–2015PR to MPA). Mice were housed in groups of 4, under a 12‐hr light/12‐hr dark cycle at 21°C, with food and water *ad libitum*. Wild‐type mice of the background strain C57/BL10 were used for gene expression profiling and induction of demyelination (see below for further details). Adult Pdgfra‐CreER^T2^:Rosa26R‐YFP mice (gift from Professor William D. Richardson, UCL, UK (Rivers et al., 2008) and Gpr17‐iCreERT2xCAG‐eGFP mice (Viganò et al., 2016) were, respectively, maintained and bred at the University of Portsmouth and University of Milan facilities; offspring were ear punched and genotyped using PCR, as previously reported (Rivers et al., 2008; Viganò et al., 2016), and mice of the correct phenotype and age (see below for the ages used) were injected intraperitoneally (i.p.,) twice a day for 5d with tamoxifen (0.1 ml of a 10 mg/ml solution, prepared in ethanol and corn oil), to induce Cre recombination and reporter expression, and brains were examined 10 days after the last injections (see below). The experiments were designed in compliance with the ARRIVE guidelines and no mice were excluded from analyses and experimental groups contained a spread of sexes. Control groups were included in all experiments, applying randomising procedures and double‐blinded analysis when possible.

### 4.2 Immunohistochemistry

For immunohistochemistry, mice were perfusion fixed intracardially under terminal anaesthesia with 4% paraformaldehyde (PFA). Brains were then dissected free and immersion fixed in 4% PFA overnight. After fixation, tissues were washed 3 times in PBS and stored at 4°C in PBS containing 0.05% NaN3 (Sigma) until use. Coronal brain sections were cut using a vibratome (Leica) at a thickness of 60 μm and used immediately or stored at 4°C in PBS/NaN3 in 24 well plates until use. Sections were treated for a blocking stage of either 10–20% normal goat serum (NGS), normal donkey serum (NDS) or 0.5% bovine serum albumin (BSA) for 1–2 h, depending on the primary antibodies to be used. Sections were washed 3 times in PBS and incubated overnight in primary antibody diluted in blocking solution containing Triton‐X (0.4%): chicken anti‐YFP (1:100, Abcam), rabbit anti‐NG2 (1:500, Millipore), rabbit anti‐Gpr17 (1:00, Cayman Labs), mouse anti‐APC/CC1 (1:700, Calbiochem), rat anti‐MBP (1:300, Millipore), rabbit‐Olig2 (1:400, Millipore), goat anti‐PDGFRα (1:400, R&D). Sections were then washed 3 times in PBS and incubated with the appropriate secondary antibody (Alexa fluor^®^‐488, Alexa fluor^®^‐568, Alexa fluor^®^‐594, Alexa fluor^®^‐647) diluted in blocking solution for 1–2 h at room temperature. Following secondary antibody incubation, tissues were washed 3 times with PBS before being mounted on glass slides using Fluoromount‐G (Southern Biotech). Detection of EdU (5‐ethynyl‐2’‐deoxyuridine) was performed as per the manufacturer's guidelines using Click‐it EdU Alexa Fluor 488 imaging kit (Invitrogen).

### 4.3 Imaging and analysis

Images were captured using a Zeiss Axiovert LSM 710 VIS40S confocal microscope and maintaining the acquisition parameters constant to allow comparison between samples within the same experiment. Acquisition of images for cell counts was done with x20 objective. Cell counts were performed in a constant field of view (FOV) of 100 µm × 100 µm or 200 µm × 200 µm, depending on the area analysed, in projected flattened images from z‐stacks formed by 10 or 15 *z*‐single plain images with 1 µm interval between them; cell density was calculated as the number of cells divided by the area of the region analysed. All data were expressed as Mean ± SEM and tested for significance using unpaired t tests.

### 4.4 RNA‐seq

For gene profiling, cerebral hemispheres from 1‐ and 18‐month‐old C57/BL10 mice were removed (n = 3 mice from each age), maintaining strict RNAase‐free and sterile conditions throughout. RNA was extracted and processed using an RNeasy Micro kit (Qiagen), after which samples were stored at −80°C until use. RNA was quantified using RiboGreen (Invitrogen) on the FLUOstar OPTIMA plate reader (BMG Labtech) and the size profile and integrity analysed on the Agilent2200 RNA ScreenTape). Input material was normalised prior to library preparation. mRNA was selected using NEBNext^®^ Poly(A) mRNA Magnetic Isolation Module (E7490S) and library preparation was completed using NEBNext^®^ Ultra Directional RNA Library Prep Kit for Illumina^®^ (E7420L, New England Biolabs), following manufacturer's instructions. Individual libraries were quantified using Qubit, and the size profile was analysed on the 2200. Next, individual libraries were assessed, using the agilent 2200 bioanalyser for quality and quantity, and then normalised and pooled together accordingly. The pooled library was denatured and loaded onto the HiSeq platform sequencer for paired‐end sequencing.

### 4.5 Meta‐analysis of generated datasets

Normalised datasets generated by RNA‐seq were analysed with EdgeR using standard pipeline methods. Differential expression analysis datasets were further processed as described previously (Rivera & Butt, 2019), using ConsensusPathDB, STRING V10 (Herwig et al., 2016; Szklarczyk et al., 2015) and STITCH v5.05 (Kuhn et al., 2008). Normalised genes dysregulated in ageing were compared to cell‐specific gene databases for OPC and MOL, using multiple published datasets (Zhang et al., 2014). The top 25 most significant genes associated with ageing in oligodendroglia are presented as a heatmap by ranking via FDR and relative fold change; the heatmap was constructed in R/Studio using the ggplot2 package.

### 4.6 SPIED/CMAP analysis

SPIED (Searchable Platform‐Independent Expression Database) and CMAP (Connectivity MAP) were used to identify small molecules predicted to induce gene signatures as the age‐sensitive OPC genes (http://spied.org.uk/cgi‐bin/HGNC‐SPIEDXXX.cgi), as described previously (Azim et al., 2017; Williams, 2013). A meta‐analysis was performed using cluster‐specific OPC genes from recent publicly available single‐cell profiling of young adult CC derived cells (https://github.com/kasumaz/AdultOLgenesis). The gene list was converted to hub genes/proteins for correlating master regulators within the totality of drug‐induced genes in the SPIED database, an additional feature of the SPIED webtool (http://spied.org.uk/cgi‐bin/HGNC‐SPIEDXXX.cgi). In this approach, small molecules generated to recapitulate transcriptional signatures associated with stimulating young adult‐associated transcriptional networks in OPCs. Gene lists were merged and CMAP interrogated for specifying pro‐OPC small molecules and their target genes were assembled into a matrix in R/RStudio with the input genes as Boolean values, and correlative scores. The R package PCAtools was used to construct a PCA plot with ggplot2 for colour and size grading using the CMAP output Pearson's scorings and numbers of target genes for each drug, respectively. The R package g:profiler was used to convert mouse to human to human gene symbols. Target genes of LY294002 were visualised using the webtool Enrichr (https://amp.pharm.mssm.edu/Enrichr/).

### 4.7 Induction of demyelination

Mice aged 6 months were deeply anaesthetised under isofluorane and a cannula (Alzet, Brain infusion kit 3) was implanted at the coordinates Bregma −0.5 mm, lateral 1 mm, depth 2.5 mm, for intraventricular infusion of agents (Azim et al., 2017). Three days following implantation, mice were anaesthetised under isofluorane and 2% lysolecithin (LPC, L‐α‐lysophosphatidylcholine; Sigma‐Aldrich) in a volume of 2 μl or sterile vehicle (saline/DMSO) was injected through the cannula to induce demyelination in the CC, as described previously (Azim & Butt, 2011). At 5 days post‐injection (DPI) of LPC, the PI3 K/Akt inhibitor LY294002, or sterile vehicle (DMSO in saline) in controls, was delivered into the cerebrospinal fluid (CSF) for 4 days via the implanted cannula, using an osmotic minipump (10 μl/h, model 1003D; Alzet Osmotic Pumps); LY294002 was administered to provide a final concentration of 2 µM in the CSF, correcting for dilution in the ventricular volume, as described previously (Azim & Butt, 2011). To measure cell proliferation, mice were given an i.p. injection of EdU (5‐ethynyl‐2′‐deoxyuridine; Click‐it EdU Alexa Fluor 488 imaging kit, Invitrogen) at 5 and 6 days DPI at 50 mg/kg. Brains were analysed at 10 DPI for gene expression and 14 DPI for cell analysis.

### 4.8 qPCR

For real‐time qPCR, total RNA was extracted as above in 4.4, from whole cerebrum to determine age‐related changes in *Gpr17* expression. To determine the effects of LY294002 on demyelination, as described above in 4.7, coronal brain sections of 500 µm thickness were cut using an adult mouse brain matrix and the CC was microdissected then flash‐frozen (analyses were performed on n = 3/4 samples, each of 2 pooled CC), as described previously (Azim et al., 2017). Maintaining strict RNAase‐free and sterile conditions throughout, total RNA extracted from these samples was reverse transcribed (RT) by Precision Nanoscript2 (PrimerDesign, UK), following manufacturer instructions, and real‐time qPCR was performed using Precision Plus qPCR Master Mix (Primer Design, UK), following manufacturer instructions, and adding to a 20 μl of PCR reaction: 10 μl of Precision Plus qPCR Master Mix, 1 μl of primer, 25 ng of Template and 4 µl of RNAse/DNAse free Water (Gibco). Amplification was performed using a Roche Lightcycler 96 according to the manufacturer's protocol. Data normalisation was performed using the housekeeping genes *Gapdh* and *Rpl13a* and expressed as relative gene expression using the 2ΔΔ‐CT method. Assays on all samples were performed in duplicate. Primer sequences are provided in Table S1.

### 4.9 Statistical analysis

All statistical analyses were performed using GraphPad Prism version 8.0 (GraphPad Software, San Diego, CA, USA) software. Data were expressed as mean ± standard error of the mean (SEM). The groups were compared using two‐tailed Student's t test where appropriate and *p* < 0.05 was considered as statistically significant.

## CONFLICT OF INTERESTS

Prof Arthur Butt and Dr Andrea Rivera are shareholders and co‐founders of the company GliaGenesis. All authors declare no other conflicts.

## AUTHOR’S CONTRIBUTIONS

ADR: Conceptualisation; Formal Analysis; Investigation; Methodology; Writing ‐ original draft; Writing ‐ review & editing. FP, IC‐D‐L‐R: Formal Analysis; Investigation; Methodology; Validation. DL: Investigation; Methodology; Writing ‐ review & editing. MPA: Writing ‐ review & editing. AMB, KA: Conceptualisation; Data curation; Formal analysis; Funding acquisition; Project administration; Resources; Supervision; Validation; Visualisation; Writing ‐ original draft; Writing ‐ review & editing.

## Supporting information

Fig S1

Fig S2

Fig S3

Table S1

## Data Availability

The transcriptomic datasets that support the findings of this study are available in the following link: https://uni‐duesseldorf.sciebo.de/s/72mMgxe40W6iTFS and upon acceptance will be placed in the Github repository. All other data that support the findings of this study are available from the corresponding authors upon reasonable request.
