# Supplementary figures and images for: Functional genomic analyses highlight a shift in Gpr17‐regulated cellular processes in oligodendrocyte progenitor cells and underlying myelin dysregulation in the aged mouse cerebrum
Source: Aging Cell. 2021 Mar 5;20(4):e13335. doi: 10.1111/acel.13335 (PMC8045941; doi:10.1111/acel.13335)

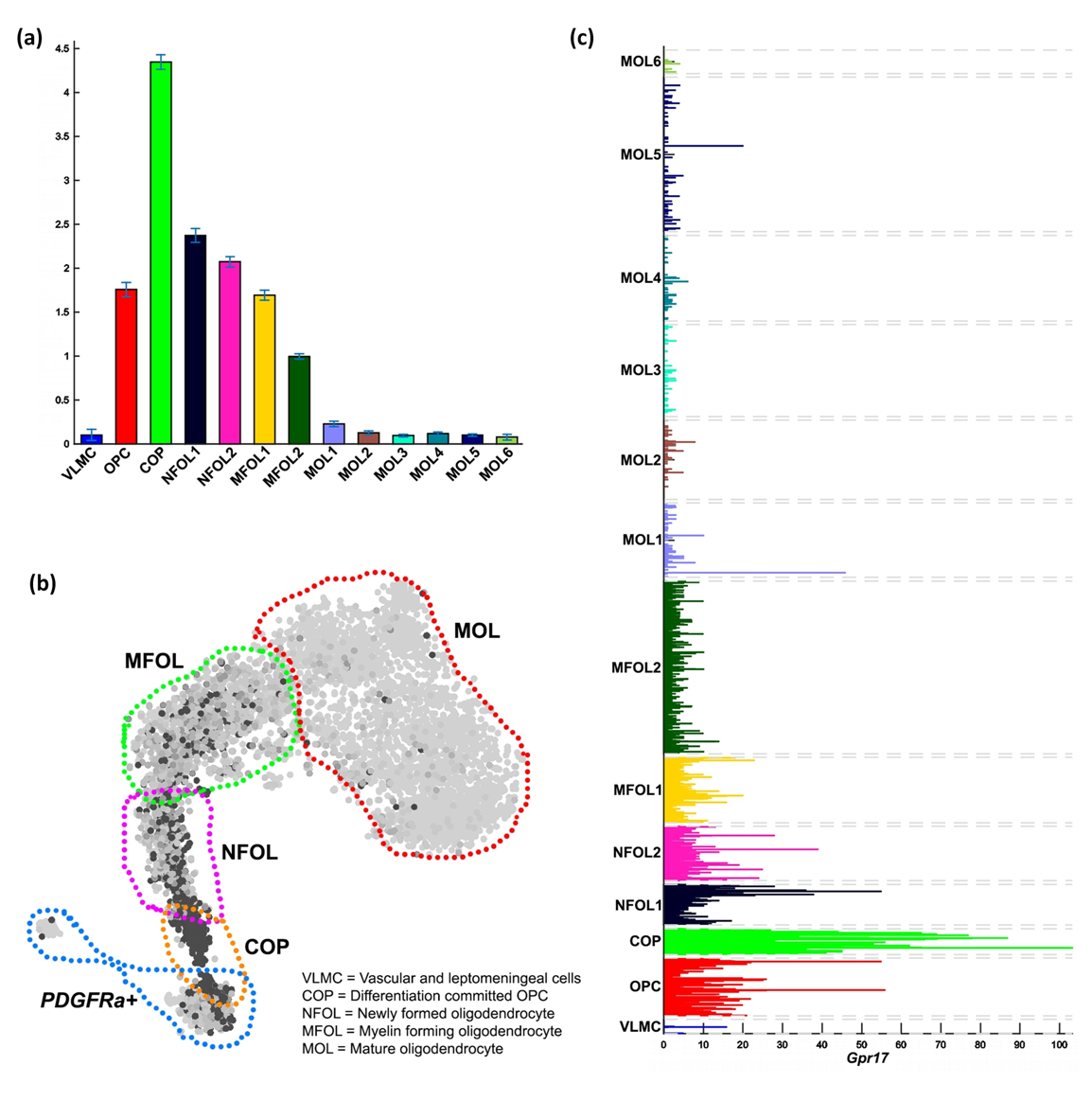

Supplement: Supplementary file 1 — Fig S1 [file ACEL-20-e13335-s001.tif]

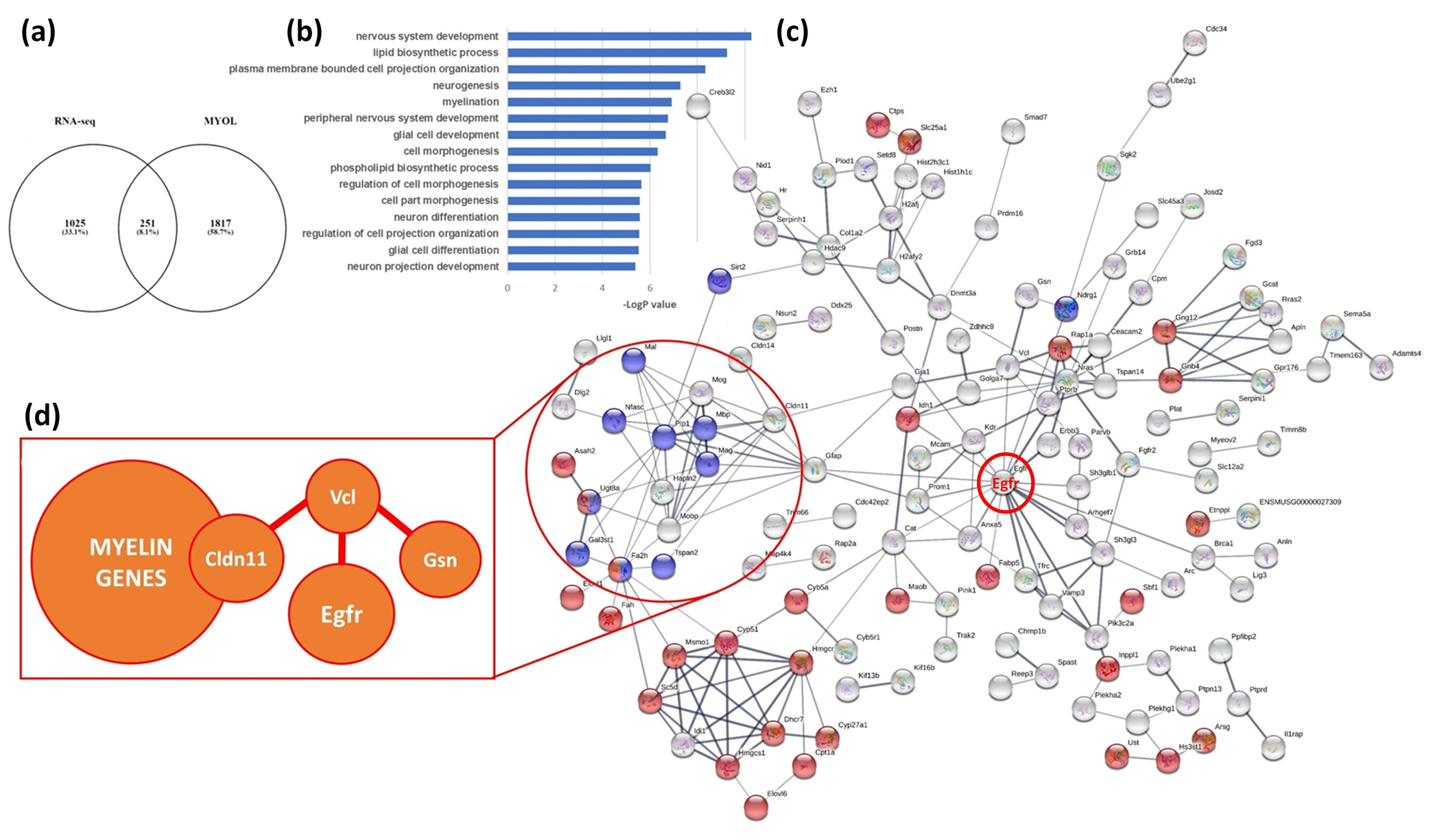

Supplement: Supplementary file 2 — Fig S2 [file ACEL-20-e13335-s003.tif]

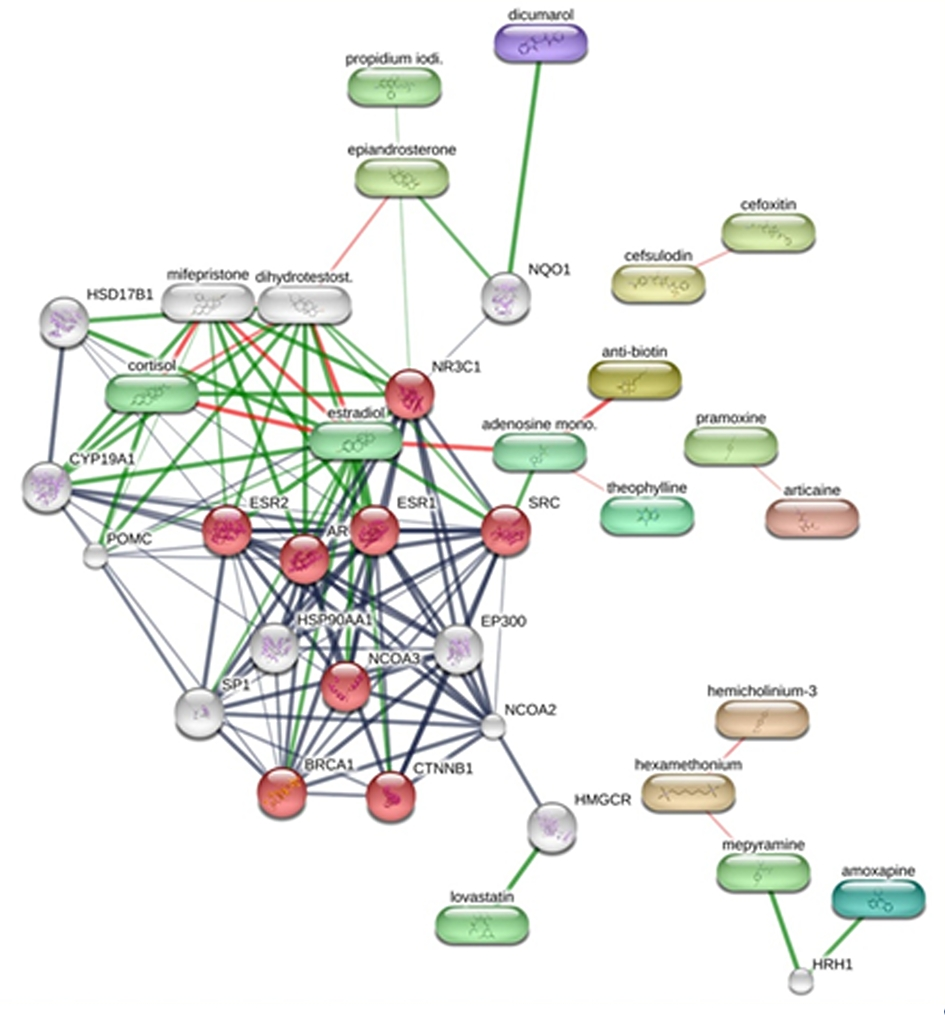

Supplement: Supplementary file 3 — Fig S3 [file ACEL-20-e13335-s004.tif]
