# Supplementary material for: Functional genomic analyses highlight a shift in Gpr17‐regulated cellular processes in oligodendrocyte progenitor cells and underlying myelin dysregulation in the aged mouse cerebrum
Source: Aging Cell. 2021 Mar 5;20(4):e13335. doi: 10.1111/acel.13335 (PMC8045941; doi:10.1111/acel.13335)
Supplement: Supplementary file 4 — Table S1 [file ACEL-20-e13335-s002.docx]

|  |  |  |  |  |
| --- | --- | --- | --- | --- |
|  | **Gene** | **Forward** | **Reverse** |  |
|  | ***Axin 2*** | 5’CTACTGTCACCCACGAAAGGCA3' | 3’GGCACGCAGAGGTGAAGTAATC5’ |  |
|  | ***Bax*** | 5’CTGGTCATGTGTGGCTTTGTCC3’ | 3’GCCAGGATCAACATGAGCTTCC5’ |  |
|  | ***Bcl2*** | 5’CCTGTGGATGACTGAGTACCTG3’ | 3’AGCCAGGAGAAATCAAACAGAGG5' |  |
|  | ***Bmp4*** | 5’CTCGACCAGGTTCATTGCAG3’ | 3’ATGGCACTACGGAATGGCTC5’ |  |
|  | ***Ccnd1*** | 5’CTGGATGCTGGAGGTCTGTGAGG3’ | 3’CTGCAGGCGGCTCTTCTTCAAG5’ |  |
|  | ***CenpaA*** | 5’GAAGTTCAGCCGTGGTGTGGAT3’ | 3’GCGTCCTCAAAGAGGTGGATGA5’ |  |
|  | ***Cntf*** | 5’AGCAAGGAAGATTCGTTCAGACC3’ | 3’TCTGCCTCAGTCATCTCACTCC5’ |  |
|  | ***Dll3*** | 5’CCAGCACTGGATGCCTTTTACC3’ | 3’ACCTCACATCGAAGCCCGTAGA5’ |  |
|  | ***Gapdh*** | 5’TTGATGGCAACAATCTCCAC3’ | 3’CGTCCCGTAGACAAAATGGT5’ |  |
|  | ***Igf1*** | 5’CACTCATCCACAATGCCTGT3’ | 3’TGGATGCTCTTCAGTTCGTG5’ |  |
|  | ***Jag1*** | 5’TGCGTGGTCAATGGAGACTCCT3’ | 3’TCGCACCGATACCAGTTGTCTC5’ |  |
|  | ***Mbp*** | 5’ATTCACCGAGGAGAGGCTGGAA3’ | 3’TGTGTGCTTGGAGTCTGTCACC5’ |  |
|  | ***Pdgfra*** | 5’AGAAAATCCGATACCCGGAG3’ | 3’AGAGGAGGAGCTTGAGGGAG5’ |  |
|  | ***Plp1*** | 5’GGGCCCCTACCAGACATCTA3’ | 3’TCCTTCCAGCTGAGCAAAGT5’ |  |
|  | ***Stat1*** | 5’ATTCACCGAGGAGAGGCTGGAA3’ | 3’TGTGTGCTTGGAGTCTGTCACC5’ |  |
|  | ***Gpr17*** | 5’GACAGGAAGGTTAGTCTCGTGA3’ | 3’TCCGTCCATGCCATATGCC5’ |  |
|  | ***Gapdh*** | 5’GTGGAGTCATACTGGAACATGTGA3’ | 3’AATGGTGAAGGTCGGTGTG5’ |  |
|  | ***Rpl13a*** | 5’CAGTGCGCCAGAAAATGC3’ | 3’GAAGGCATCAACATTTCTGGAA5’ |  |
|  |  |  |  |  |

**Table 1 Primer Sequences**
